# STAT3 can be activated through paracrine signaling in breast epithelial cells

**DOI:** 10.1186/1471-2407-8-302

**Published:** 2008-10-21

**Authors:** Jacqueline C Lieblein, Sarah Ball, Brian Hutzen, A Kate Sasser, Huey-Jen Lin, Tim HM Huang, Brett M Hall, Jiayuh Lin

**Affiliations:** 1 Department of Pediatrics, The Ohio State University, Columbus, USA; 2 Ohio State Biochemistry Program, The Ohio State University, Columbus, USA; 3 Molecular, Cellular, and Developmental Biology Program, The Ohio State University, Columbus, USA; 4 Division of Medical Technology, School of Allied Medical Professions, The Ohio State University, Columbus, USA; 5 Human Cancer Genetics Program, The Ohio State University, Columbus, USA; 6 Department of Molecular Virology, Immunology, and Medical Genetics, The Ohio State University, Columbus, USA; 7 Experimental Therapeutics Program, The Ohio State University Comprehensive Cancer Center, The Ohio State University, Columbus, USA; 8 The Research Institute at Nationwide Children's Hospital, The Ohio State University, WA5020 Research Building II, 700 Children's Drive, Columbus, OH 43205, USA

## Abstract

**Background:**

Many cancers, including breast cancer, have been identified with increased levels of phosphorylated or the active form of Signal Transducers and Activators of Transcription 3 (STAT3) protein. However, whether the tumor microenvironment plays a role in this activation is still poorly understood.

**Methods:**

Conditioned media, which contains soluble factors from MDA-MB-231 and MDA-MB-468 breast cancer cells and breast cancer associated fibroblasts, was added to MCF-10A breast epithelial and MDA-MB-453 breast cancer cells. The stimulation of phosphorylated STAT3 (p-STAT3) levels by conditioned media was assayed by Western blot in the presence or absence of neutralized IL-6 antibody, or a JAK/STAT3 inhibitor, JSI-124. The stimulation of cell proliferation in MCF-10A cells by conditioned media in the presence or absence of JSI-124 was subjected to MTT analysis. IL-6, IL-10, and VEGF levels were determined by ELISA analysis.

**Results:**

Our results demonstrated that conditioned media from cell lines with constitutively active STAT3 are sufficient to induce p-STAT3 levels in various recipients that do not possess elevated p-STAT3 levels. This signaling occurs through the JAK/STAT3 pathway, leading to STAT3 phosphorylation as early as 30 minutes and is persistent for at least 24 hours. ELISA analysis confirmed a correlation between elevated levels of IL-6 production and p-STAT3. Neutralization of the IL-6 ligand or gp130 was sufficient to block increased levels of p-STAT3 (Y705) in treated cells. Furthermore, soluble factors within the MDA-MB-231 conditioned media were also sufficient to stimulate an increase in IL-6 production from MCF-10A cells.

**Conclusion:**

These results demonstrate STAT3 phosphorylation in breast epithelial cells can be stimulated by paracrine signaling through soluble factors from both breast cancer cells and breast cancer associated fibroblasts with elevated STAT3 phosphorylation. The induction of STAT3 phosphorylation is through the IL-6/JAK pathway and appears to be associated with cell proliferation. Understanding how IL-6 and other soluble factors may lead to STAT3 activation via the tumor microenvironment will provide new therapeutic regimens for breast carcinomas and other cancers with elevated p-STAT3 levels.

## Background

After heart disease and lung cancer, breast cancer ranks as the third leading cause of death in women in the United States, accounting for over 40,000 mortalities in 2006 [1]. Moreover, the American Cancer Society estimates that around 178,000 invasive breast cancer cases will be found in 2007 [1]. Much advancement has been made in breast cancer treatment, including the characterization of breast disease based on the patient's hormone receptor status, understanding the role of inherited genetic abnormalities, and the assessment of the risk for invasive disease [2,3]. Moreover, understanding the molecular basis for each patient's disease has allowed for more effective treatment. Although mortality rates are declining, it is clear that with over 40,000 deaths each year there is still much to learn about the disease and how to best increase each patient's chance for survival. There are still many poorly understood molecular factors which increase breast tumorigenesis. Among these are the Signal Transducers and Activators of Transcription (STAT) proteins. Constitutive activation of STAT proteins is found in an astounding number of breast cancers and other human diseases [4-6]. It is clear that we need to understand the role of STAT proteins in order to find more effective and personalized treatments for breast cancer patients.

STAT proteins constitute a family of transcription factors which lead to the downstream activation of various genes involved in cell growth, differentiation, and survival [7]. These proteins exist as inactive monomers in the cytoplasm and become activated upon tyrosine phosphorylation. This phosphorylation event allows the STAT molecules to form homodimers or heterodimers with other activated STAT family members via its Src-homology 2 (SH2) domain [8]. This dimer can then enter the nucleus and activate transcription of various genes [9,10]. There have been seven mammalian STAT genes identified to date [8,11]. The STAT3 protein is one of the major members of this family that has been widely implicated in numerous cancers [7,12]. Activation of STAT3 can lead to cell-cycle progression, anti-apoptotic effects, proangiogenesis, immune evasion, and tumor invasion and metastasis [5,7]. These characteristics incorporate many of the hallmarks of cancer [13]. In addition, STAT3 has been implicated in the activation of downstream cytokines, including vascular endothelial growth factor (VEGF), which may also contribute to tumorigenesis [14].

Constitutive activation of STAT3 has been reported to be sufficient to induce tumor formation in a range of human cancers [12,15]. In addition, constitutively activated STAT3 is frequently found in breast cancer cell lines and patients with advanced breast disease, but is absent in normal breast epithelial cells [16-18]. In particular, the phosphorylated form of STAT3 at tyrosine residue 705 (Y705) is frequently found elevated within breast carcinomas [16]. Receptor-mediated activation of STAT proteins, especially of STAT3 and STAT5, has been found to occur both *in vitro *and *in vivo *in breast carcinogenesis [19]. Therefore, understanding the events leading to STAT3 activation will provide critical insight for treating and preventing breast tumorigenesis.

The tumor microenvironment, or how tumor cells cross-talk with other components in their surroundings, is a vital feature of emerging cancer research. Paracrine signaling within the microenvironment is important for regulation of such characteristics as proliferation, differentiation, and apoptosis [20]. The stroma also plays a role in promoting angiogenesis, and altering the extracellular matrix, as well as increasing inflammatory cell recruitment to the area [21-23]. Fibroblasts are a significant component of the stroma and have been identified to have an active role in tumorigenesis, including the regulation of breast cancer cell growth [24-26].

Recent research has demonstrated that activated STAT3 plays an important role in the cross-talk between cancer cells and immune cells [27]. However, little has been known about the activation of STAT3 in regards to the other components within the tumor microenvironment, including the interaction between tumor cells and surrounding fibroblasts and epithelial cells.

Our laboratory is examining the effects of paracrine signaling from breast cancer cells and breast fibroblasts with increased phosphorylated STAT3 (p-STAT3) levels on tumor progression. We show here that soluble factors released from breast cell lines with high levels of STAT3 phosphorylation are sufficient to confer increased p-STAT3 levels in recipient breast cell lines with low activation of this protein. This activation occurs through the JAK/STAT3 signaling pathway, and mediates phenotypic changes in the treated cells, including enhanced cell proliferation and transformation of the cells to increase production of cytokines, which may promote tumor formation. Importantly, we noticed a correlation between increased levels of p-STAT3 and Interleukin-6 (IL-6) production from cells with constitutively active p-STAT3, and found that IL-6 is one of the major factors contributing to the paracrine activation of STAT3 in breast cell lines without upregulated p-STAT3. Understanding how IL-6 and other soluble factors are involved in this paracrine activation of STAT3 via the tumor microenvironment may provide new therapeutic insight to treat breast carcinomas and other cancers with elevated p-STAT3 and IL-6 levels.

## Methods

### Cell Culture and co-culture

MDA-MB-453, MDA-MB-468, MDA-MB-231 human breast cancer cell lines and MCF-10A mammary epithelial cells were purchased from ATCC. The breast cancer associated fibroblasts were provided by Dr. Tim Huang at the Ohio State University Medical Center, which were derived from breast cancer patients and the fibroblastic characteristics were confirmed by the presence of their hallmark antigens: vimentin and prolyl-4-hydroxylase (Data not shown). Human breast cancer associated fibroblasts, MDA-MB-453, MDA-MB-468, and MDA-MB-231 breast cancer cell lines were maintained in 10% fetal bovine serum (FBS) (Invitrogen), Dulbecco's Modification of Eagle's Medium (DMEM), 1× with 4.5 g/L, L-glutamine, & sodium pyruvate (Mediatech) with 1% Penicillin/Streptomycin (P/S). MCF-10A mammary epithelial cell line was grown in Ham's F12 media (Mediatech) with 5 μg/ml insulin, 1 μg/ml hydrocortisone, 10 μg/ml epidermal growth factor (EGF), 100 μg/ml cholera toxin, 5% FBS, and 1% P/S. MDA-MB-453 cells were seeded in 6-well plates and MDA-MB-468 cells were grown on top of 0.22 μM filter inserts to block physical contact between two different cell lines. MDA-MB-453 cells were co-cultured with the MDA-MB-468 cells in 5% FBS, 1× DMEM for 4 days after which the MDA-MB-453 cell lysates were collected for Western blot analysis.

### Conditioned Media

MDA-MB-468 or MDA-MB-231 cell lines were grown in two, T-75 flasks. Breast fibroblasts were grown in four, 10-cm plates. At 70% confluency, the cells were refed with 2% FBS, 1× DMEM media for 48 hours. The conditioned media that was in the presence of p-STAT3-cell lines (MDA-MB-468, MDA-MB-231 or breast fibroblasts) was filtered (0.22 μM) to remove any cellular debris. The conditioned media containing soluble factors secreted from a p-STAT3-positive cell line was then used to treat MDA-MB-453 or MCF-10A cells in 10-cm plates for various time periods between 0.5 and 24 hours. MDA-MB-453 and MCF-10A cell lysates were collected at their designated time points for Western blot analysis.

### Western blot analysis

**Figure 1:**
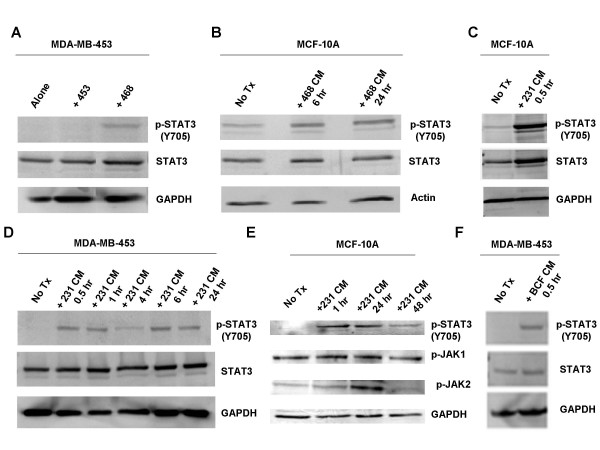
**Soluble factor(s) secreted by MDA-MB-231, MDA-MB-468, and breast cancer associated fibroblasts are sufficient to amplify p-STAT3 (Y705) levels in MDA-MB-453 and MCF-10A.** A. MDA-MB-453 cells were co-cultured with MDA-MB-468 cells for 120 hours. B. MCF-10A cells were treated with MDA-MB-468 conditioned media for 6 and 24 hours. C. MCF-10A cells were treated with MDA-MB-231 conditioned media for 0.5 hours. D. MDA-MB-453 cells were treated with MDA-MB-231 conditioned media for various time points between 0.5 and 24 hours. E. MCF-10A cells were treated with MDA-MB-231 conditioned media for 1, 24, and 48 hours. F. MDA-MB-453 cells were treated with conditioned media from breast cancer associated fibroblasts for 0.5 hours. 453: MDA-MB-453; 468: MDA-MB-468; 231: MDA-MB-231; BCF: breast cancer associated fibroblasts; No Tx: no treatment; CM: conditioned media; hr: hour.

For MDA-MB-453, 100 μg of total protein, or for MCF-10A, 60 μg of total protein, from their respective cell lysates were subjected to SDS polyacrylamide gel electrophoresis (PAGE) and transferred to PVDF membrane. Membranes were blotted with phospho-specific STAT3 antibody (Tyrosine 705; #9131 Cell Signaling Tech. [Beverly, MA]), which does not cross react with any other STAT protien; phospho-specific STAT3 antibody (Serine 727; #9134S Cell Signaling Tech. [Beverly, MA]), phospho-independent STAT3 antibody (#9132 Cell Signaling Tech.); phospho-specific AKT antibody (Serine 473; #9271S Cell Signaling Tech. [Beverly, MA]); phospho-specific JAK1 antibody (Tyrosine 1022/1023; #3331 Cell Signaling Tech. [Beverly, MA]), which may cross react with p-JAK2; phospho-specific JAK2 antibody (Tyrosine 1007/1008; #07-123 Upstate Biotech. [Temecula, CA]), which does not cross react with any other protein; Cyclin D1 antibody (#2922 Cell Signaling Tech. [Beverly, MA]); and GAPDH antibody (MAB374 Chemicon International, Inc. [Temecula, CA]). β-Actin antibody (A 1978 SIGMA [St. Louis, MO]) was used in Fig. 1B.

### Antibody neutralization

0.22 μM filtered conditioned media (for IL-6, LIF, and GAPDH neutralization) or adherent cells (for gp130 receptor neutralization) were treated with 4 μg/mL of antibody (IL-6: MAB206 R&D Systems [Minneapolis, MN]; LIF: AF-250-NA R&D Systems [Minneapolis, MN]; GAPDH: MAB374 Chemicon International, Inc; gp130: MAB228 R&D Systems) for one hour on a slow rotor. The conditioned media was left at 25°C during this incubation, while adherent cell lines were kept in a CO_2 _incubator at 37°C.

### MTT Cell proliferation Assay

MCF-10A cells were seeded in 96-well plates (6000 cells/well) in Ham's F12 media with the additives as described above. After 24 hours, cells were washed and media was replaced with 2% FBS, 1× DMEM. The following day, MCF-10A cells were treated with MDA-MB-231 conditioned media for various time points. MDA-MB-231 condition media was made at a concentration of 5 × 10^6 ^cells/20 ml of 2% FBS, 1× DMEM media for 48 hours. At the end of each time point, 25 μl of MTT (Thiazolyl Blue Tetrazolium Bromide: M5655, SIGMA) was added to each well of the plate and incubated for 3.5 hours. After this, 100 μl of N,N-dimethylformamide (D4551, SIGMA) solubilization solution was added to each well. Plates were left at room temperature overnight to allow complete lysis of cells, and read at 450 nm the following day.

### ELISA analysis

**Figure 2:**
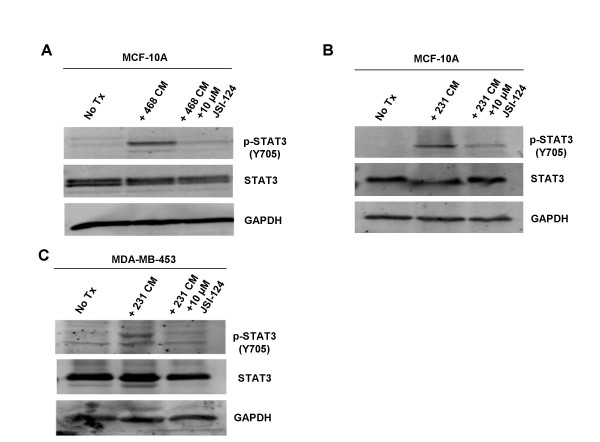
**Secretion of VEGF, IL-10, and IL-6 cytokines by various breast cell lines.** A. VEGF secretion by breast cell lines after 24 hours. VEGF production does not correlate with p-STAT3 levels. B. IL-10 secretion by breast cell lines after 24 hours. IL-10 production does not correlate with p-STAT3 levels. C. IL-6 secretion by breast cell lines after 48 hours. p-STAT3-positive cell lines secrete high levels of IL-6 and cell lines that do not express constitutive p-STAT3 have low levels of IL-6 secretion. Cell lines that do not express constitutive p-STAT3: MDA-MB-453 and MCF-10A; p-STAT3-positive cells: MDA-MB-468, MDA-MB-231, and breast cancer associated fibroblasts (BCF).

**Figure 3:**
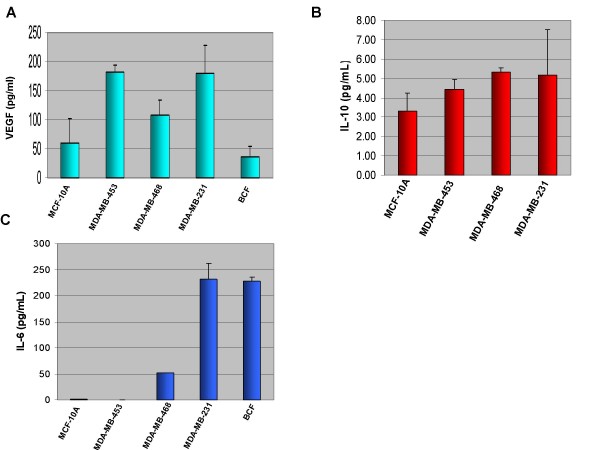
**Soluble factor(s) from MDA-MB-231 conditioned media were able to confer increased growth rates in MCF-10A.** A. MCF-10A cells were treated with MDA-MB-231 conditioned media and analyzed by MTT assay for cell viability. B. MCF-10A cells were treated with MDA-MB-231 conditioned media and/or 5 μM JSI-124. C. MCF-10A cells were treated with MDA-MB-231 conditioned media for varying amounts of time. Cell viability was analyzed by MTT assay. 231: MDA-MB-231; No Tx: no treatment; CM: conditioned media.

For Fig. 2A and Fig 2B: The cells were grown in 10-cm plates until they had reached 75–80% confluency. Their media was then replaced with DMEM supplemented with 2% FBS. These cells were then incubated at 37°C for an additional 24 hours, whereupon their media was collected, filter-sterilized, aliquoted, and stored at -80°C. For Fig. 3, cells were grown in 6-well plates until they reached 75–80% confluency. Their media was then replaced with DMEM supplemented with 2% FBS for 24 hours. The cells were then treated with MDA-MB-231 cells' conditioned media for 24, 48, or 96 hours. After this treatment, the MCF-10A cells were washed and the media was replaced with DMEM supplemented with 2% FBS. The cells were then allowed to secrete their own cytokines for 24 hours, after which the media was collected, filter-sterilized, aliquoted, and stored at -80°C. Secretion of IL-6 and IL-10 was quantified with ELISA kits obtained from Diaclone (catalog #950.030.096, and 950.060.096, respectively, [Stamford, CT]); VEGF secretion was monitored with an ELISA kit from R&D Systems (catalog #DVE00, [Minneapolis, MN]). Each assay was performed per the manufacturer's instructions. Samples were assayed in triplicate. Error bars represent +/- one standard deviation. For Fig. 2C: The cells were plated in triplicate at a concentration of 30,000 cells/well in 2% FBS, 1× DMEM in a 96-well plate. Cells were grown for 48 hours, the media was removed, filtered, and secretion of IL-6 was assayed using a human IL-6 ELISA kit obtained from Diaclone (catalog #950.030.096). The manufacturer's protocol was also used for this assay.

### RT-PCR analysis

MCF-10A cells were grown to 80% confluency in six-well plates and then switched to DMEM supplemented with 2% FBS for 24 hours. The media was then replaced with conditioned media from MDA-MB-231 or plain 2% FBS DMEM for the listed times, after which RNA was extracted with the Qiagen RNeasy kit (Qiagen, Valencia, CA) and stored at -80°C. Sample RNA was reverse transcribed using Omniscript RT (Qiagen), and 2 μl of the cDNA products were used to carry out non-quantitative RT-PCR. Primers for human IL-6 were 5'-TACCCCCAGGAGAAGATTCC-3' (forward) and 5'-TTTCAGCCATCTTTGGAAGG-3' (reverse). Primers for GAPDH were 5'-ATGGGGAAGGTGAAGGTCG-3' (forward) and 5'-GGGGTCATTGATGGCAACAATA-3' (reverse). PCR amplifications were performed as follows: 5 min at 94°C followed by 30 cycles of (30 sec at 94°C, 30 sec at 55°C, 30 sec at 72°C) and a final extension at 72°C for 5 min. The PCR products were then run on 2% agarose gels, stained with ethidium bromide and visualized under UV light.

## Results

### Soluble Factor(s) Secreted by MDA-MB-231, MDA-MB-468, and Breast Cancer Associated Fibroblasts are Sufficient to Amplify p-STAT3 (Y705) Levels within MDA-MB-453 and MCF-10A Breast Epithelial Cells

Since the role of the tumor microenvironment on STAT3 activation is not well understood, it was of interest to examine the effects of paracrine signaling from breast cancer cells containing constitutively activated STAT3 (high levels of p-STAT3) on breast cancer cells without constitutively activated STAT3. MDA-MB-453, an estrogen receptor alpha negative (ERα-) breast cancer cell line with undetectable levels of p-STAT3 (Y705) was co-cultured with MDA-MB-468, an ERα- breast cancer cell line with increased levels of p-STAT3. Soluble factors secreted from the MDA-MB-468 cell line were sufficient to increase STAT3 phosphorylation in MDA-MB-453 after five days of growth in co-culture (Fig. 1A). To determine if this was a phenomena only observed with MDA-MB-453, we treated an immortalized, non-cancerous breast epithelial cell line, MCF-10A, which expresses undetectable levels of p-STAT3 (Y705), with media conditioned in the presence of MDA-MB-468 cells for 24 hours. This conditioned media, containing soluble factors from MDA-MB-468 cells, was also sufficient to increase p-STAT3 in MCF-10A cells after 6 and 24 hours of treatment (Fig. 1B).

Further experiments were performed to support these results. Conditioned media from an aggressive ERα- breast cancer cell line with high p-STAT3 (Y705), MDA-MB-231, was used to treat MCF-10A and MDA-MB-453 cell lines. Similar results were obtained, as the soluble factors secreted by MDA-MB-231 cells in this media also induces an increase of p-STAT3 in both MCF-10A and MDA-MB-453 cell lines (Fig. 1C, 1D, and 1E). Increased levels of p-STAT3 were detectable as early as 30 minutes after conditioned media treatment, persisting through at least 24 to 48 hours (Fig. 1D and 1E).

Additionally, we used breast cancer associated fibroblasts (BCF) with high p-STAT3 levels to treat MDA-MB-453 cells, demonstrating that soluble factors from BCF could also induce STAT3 in MDA-MB-453 cells as early as 30 minutes (Fig. 1F). Furthermore, serially diluting the conditioned media in fresh media before treatment of cells demonstrated a proportional decline of p-STAT3 signal (data not shown). This suggests that the increase in p-STAT3 levels is directly correlated with the soluble factors contained within the conditioned media. Phosphorylation of STAT3 at the tyrosine residue is necessary for STAT3 activation [28]. There is also another phosphorylation site at the Serine 727 residue in the transactivation domain of STAT3, which may lead to full activation of STAT3, although serine phosphorylation has no influence on STAT3's ability to bind DNA [29]. We did not see any difference in phosphorylation of the Serine 727 residue after conditioned media treatment (data not shown). In addition, we assayed for phosphorylated AKT (p-AKT) at Serine 473 (S473) after treatment of MCF-10A with MDA-MB-468 cells' conditioned media; however, no elevation of p-AKT (S473) was observed under these same conditions (data not shown).

### Activation of STAT3 Occurs through the JAK/STAT3 Signaling Pathway

**Figure 4:**
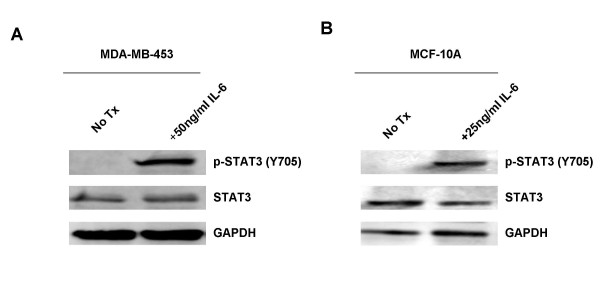
**Activation of STAT3 occurs through the JAK/STAT3 signaling pathway.** A. MCF-10A cells were treated with MDA-MB-468 conditioned media for 0.5 hours. Treatment with 10 μM JSI-124 one hour prior to treatment with conditioned media reduced STAT3 activation. B. MCF-10A cells were treated with MDA-MB-231 conditioned media for 0.5 hours. Treatment with 10 μM JSI-124 one hour prior to treatment with conditioned media reduced STAT3 activation. C. MDA-MB-453 cells were treated with MDA-MB-231 conditioned media for 0.5 hours. Treatment with 10 μM JSI-124 one hour prior to treatment with conditioned media reduced STAT3 activation. 468: MDA-MB-468; 231: MDA-MB-231; 453: MDA-MB-453; No Tx: no treatment; CM: conditioned media.

There are multiple ways in which STAT proteins can be phosphorylated, including via Src or JAK non-receptor tyrosine kinases, or via growth factors such as epidermal growth factor (EGF) and platelet-derived growth factor (PDGF) [30]. Therefore, it was important to identify through which pathway this paracrine activation occurs. JSI-124 (Cucurbitacin I) is a highly specific inhibitor to the JAK/STAT3 pathway [31]. Pre-treating MCF-10A cells with 10 μM of JSI-124 for one hour prior to MDA-MB-468 cells' conditioned media treatment was sufficient to abolish the elevated levels of p-STAT3 (Y705) (Fig. 4A). Similar results were also obtained using the conditioned media derived from MDA-MB-231 cells (Fig. 4B). A different recipient, the MDA-MB-453 breast cell line, also responded to JSI-124 upon conditioned media treatment in a manner similar to MCF-10A (Fig. 4C). We also looked at the levels of p-JAK1 and p-JAK2 after treatment with conditioned media to see which JAK was responsible for the induction of p-STAT3. We did not find a difference in the levels of p-JAK1; however, we did see an increase in the level of p-JAK2 which corresponded to increased p-STAT3 levels. Taken together, these data suggest that this paracrine signaling occurs through the JAK/STAT3 signaling pathway and is mediated by the soluble factor(s) in the conditioned media. Nonetheless, other pathways may still play a role and need to be explored.

### p-STAT3-positive Cell Lines Secrete High Levels of IL-6 while p-STAT3-negative Cell Lines have Low Levels of IL-6 Secretion

We next attempted to determine which soluble factors in the conditioned media were responsible for this increase in p-STAT3. We performed ELISA analysis for various cytokines secreted from our cells of interest. We were interested in examining production levels of VEGF, Interleukin-10 (IL-10), and IL-6, as they have been reported as activators as well as downstream targets of STAT3 [27]. It was of significance to determine if our breast cancer epithelial cell lines and associated fibroblasts could secrete these factors, and whether these may be the paracrine activators of STAT3 observed in our experiments.

**Figure 5:**
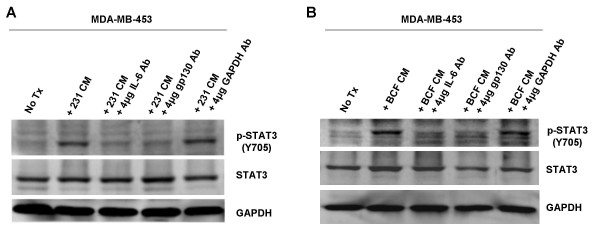
**Addition of IL-6 is sufficient to increase levels of p-STAT3 in MDA-MB-453 and MCF-10A cell lines.** A. MDA-MB-453 cells were treated with 50 ng/ml IL-6 for 30 minutes. B. MCF-10A cells were treated with 25 ng/ml IL-6 for 30 minutes.

ELISA analysis for the secretion of VEGF showed that while some p-STAT3-positive cell lines, namely MDA-MB-468 and MDA-MB-231, had moderate production of VEGF, MDA-MB-453 cells, which do not express constitutively active STAT3, also had elevated levels of VEGF secretion In addition, BCF had lower levels of VEGF secretion (Fig. 2A). Therefore, VEGF secretion does not correlate with the p-STAT3 status in these cells. Analysis of IL-10 secretion showed considerably low levels of production (ranging between 3.31 and 5.31 pg/mL, Fig. 2B) in all cell lines examined regardless their p-STAT3 status, and was thus excluded from further studies. While no correlation between high levels of p-STAT3 and the secretion of VEGF or IL-10 was observed (Fig. 2A and 2B), high levels of IL-6 secretion were seen in p-STAT3-positive cell lines: MDA-MB-231, MDA-MB-468, and BCF (Fig. 2C). Moreover, MCF-10A and MDA-MB-453 breast cell lines that do not express constitutively active STAT3, had low or negligible secretion of IL-6 (Fig. 2C). ELISA arrays for multiple cytokines were also performed to compare the difference in secretion of cytokines between MDA-MB-231 and MCF10A. These also confirmed MDA-MB-231 breast cancer cells secrete much higher levels of IL-6 than immortalized MCF-10A breast cells (data not shown). Once we had singled out IL-6 as the soluble factor that may be responsible for the paracrine activation of STAT3, we decided to treat cells that do not express constitutively active STAT3, MCF-10A and MDA-MB-453, with recombinant human IL-6 and look at the effects on STAT3. We found that the addition of IL-6 caused an increase in the levels of p-STAT3 in both cells lines after just 30 minutes of treatment (Fig. 5A and 5B). Immunohistochemical analysis published in a recent paper by Berishaj et al also revealed a strong positive correlation between the expression of IL-6 and the phosphorylation status of STAT3 [32]. These observations suggest that IL-6 is an important soluble factor in the conditioned media which may lead to the paracrine activation of STAT3 in breast cancer.

### Inhibition of IL-6 or gp130 can Block the Elevation of p-STAT3 (Y705) in MDA-MB-453 cells

**Figure 6:**
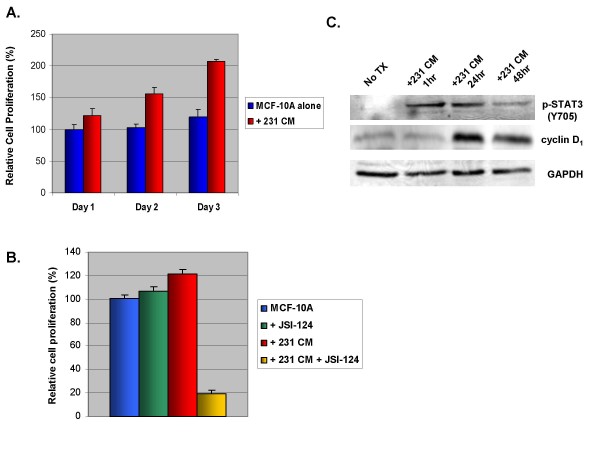
Inhibition of IL-6 or gp130 is sufficient to block increased levels of p-STAT3 (Y705) in MDA-MB-453. A. MDA-MB-231 conditioned media was subjected to 4 μg/ml IL-6, gp130, or GAPDH antibody neutralization. This neutralized conditioned media was then used to treat MDA-MB-453 cells for 0.5 hours. B. BCF conditioned media was subjected to 4 μg/ml IL-6, gp130, or GAPDH antibody neutralization. This neutralized conditioned media was then used to treat MDA-MB-453 cells for 0.5 hours. 453: MDA-MB-453; 231: MDA-MB-231; BCF: breast cancer associated fibroblasts; No Tx: no treatment; CM: conditioned media.

Since IL-6 secretion correlates with high p-STAT3 levels, it was important to determine whether blocking IL-6 would be sufficient to inhibit the increase in p-STAT3 seen after conditioned media treatment. IL-6 antibody was added to the MDA-MB-231 conditioned media in order to bind and neutralize IL-6 prior to treating MDA-MB-453 cells. Neutralizing IL-6 was sufficient to block activation of STAT3 (Fig. 6A). In addition, gp130 antibody was added to MDA-MB-453 cells to bind the gp130 component of the IL-6 receptor prior to conditioned media treatment. This was also able to block the increase of p-STAT3 (Fig. 6A). These results were consistent when repeated with fibroblasts' conditioned media on MDA-MB-453 cells (Fig. 6B). Together, these results suggest that IL-6 plays a major role in the paracrine activation of STAT3 in breast epithelial cells.

### Soluble Factor(s) Released from MDA-MB-231 Confer Accelerated Cell Proliferation in MCF-10A cells

Since elevated p-STAT3 levels were observed in MCF-10A cells after treatment with conditioned media, we next examined if this phenomena renders a transformed phenotype in MCF-10A, a non-cancerous breast epithelial cell line. Using MTT Assay, we demonstrated that soluble factors from MDA-MB-231 cells conferred amplified cell proliferation in MCF-10A cells compared to no treatment (Fig. 3A). To determine whether this increased cell proliferation is in fact mediated through the JAK/STAT3 pathway, we used JSI-124 in our MTT assays to see if we could block the enhanced growth. Interestingly, the inhibitor was able to significantly restrain the accelerated cell proliferation of MCF-10A cells treated with MDA-MB-231 conditioned media (Fig. 3B). Moreover, the conditioned media-treated cells showed increased sensitivity to JSI-124 over untreated cells. This suggests that after cells become dependent on an activated STAT3 pathway for survival or growth, shutting this pathway down selectively inhibits cell viability. Past research has showed similar cytoxicity using a dominant negative STAT3 and STAT3 inhibitors in cervical cancer cell lines expressing increased p-STAT3 [33]. This shows promise for targeting STAT3 in cancer patients with the use of a JAK/STAT inhibitor, as these results suggest reduced toxicity to normal cells without the enhanced STAT3 activation.

We decided to look at one of the downstream targets of STAT3, Cyclin D1, which is involved in the G_1_/S cell cycle transition, to explain the increased cell proliferation [6]. After treating MCF-10A cells with MDA-MB-231 conditioned media for varying amounts of time we looked to see if there was any change in cyclin D1 levels. We found increased levels after both 24 and 48 hours of treatment (Fig. 3C). This could explain the increased cell proliferation seen.

### Soluble Factor(s) from MDA-MB-231 cells Stimulate MCF-10A cells to Increase Production of IL-6

**Figure 7:**
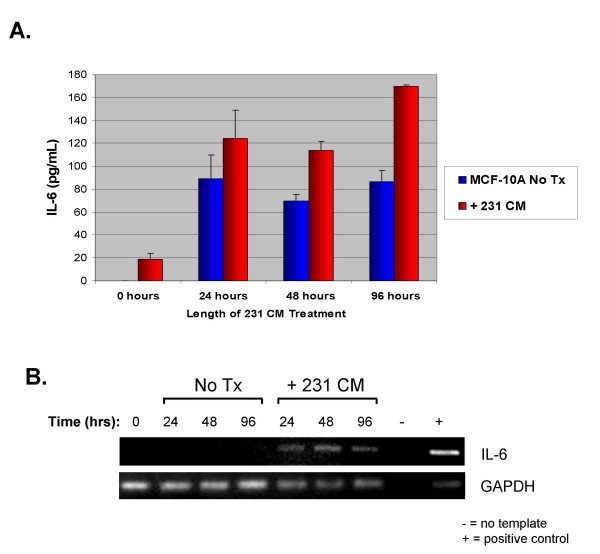
**Soluble factor(s) from MDA-MB-231 stimulate MCF-10A to increase production of IL-6.** A. MCF-10A cells were treated with MDA-MB-231 conditioned media for 24, 48, or 96 hours. After this treatment time, MCF-10A cells were washed and allowed to produce their own cytokines for 24 hours, after which ELISA analysis was performed to assay for IL-6 secretion. For the 0 hour treatment, conditioned media was added, but was washed away immediately and collected for analysis. This was done to show that any soluble factors from the MDA-MB-231 conditioned media were washed away and were not contaminating our results. For untreated MCF-10A cells, the cells were mock-treated with fresh 2% FBS, 1× DMEM instead. B. To confirm that soluble factors from the MDA-MB-231 conditioned media were inducing MCF-10A to produce IL-6, RNA collected from these cells and used to perform non-quantitative RT-PCR. RNA from MDA-MB-231 was used as a positive control. 231: MDA-MB-231; No Tx: no treatment; CM: conditioned media.

In this study, we found a positive correlation between IL-6 production and STAT3 activation in p-STAT3-positive cell lines. In addition, soluble factors, particularly those within the IL-6 family of cytokines, are important in paracrine activation of STAT3. Moreover, activation of STAT3 has been implicated in the upregulation of various cytokines, including IL-6 [27]. Therefore, we were interested in determining whether paracrine activation of STAT3 could also induce the conditioned media-treated cells to increase their production of IL-6. Since MCF-10A cells do not express constitutively activated STAT3 and secrete low levels of IL-6, we examined the possibility that soluble factors released from MDA-MB-231 breast cancer cell lines may stimulate MCF-10A cells to produce different cytokines, including the IL-6 family cytokines and others. We treated MCF-10A cells with conditioned media from MDA-MB-231 breast cancer cells, which produce considerable levels of IL-6. After various time points, the conditioned media was extensively washed away, and MCF-10A cells were allowed to produce their own IL-6 for 24 hours. IL-6 secretion was analyzed by ELISA. Our results suggest that soluble factors within the MDA-MB-231 conditioned media are also sufficient to stimulate an increase in IL-6 levels of approximately 2-fold (Fig. 7A) in MCF-10A cells compared to untreated MCF-10A cells (96 hour treatment). To confirm these results, we performed non-quantitative RT-PCR using cDNA reverse transcribed from the RNA of MCF-10A cells treated with MDA-MB-231 conditioned media or DMEM alone. Bands corresponding to the IL-6 PCR product are readily detected in the MCF-10A samples treated with conditioned media, but are otherwise absent in the untreated controls (Fig. 7B). This is the first report showing that the secretion of IL-6 can be induced by paracrine signaling in non-transformed MCF-10A mammary epithelial cells.

## Discussion

Our laboratory was interested in determining how breast cells with increased levels of p-STAT3 could influence their surroundings. Our findings show that soluble factors from breast cell lines with high levels of STAT3 activation are sufficient to confer increased p-STAT3 levels in other cell lines with low activation of this protein. We used several different cell lines with constitutive p-STAT3, including breast cancer epithelial cells and fibroblasts. In addition, paracrine activation was seen in both cancerous and non-tumorigenic human mammary epithelial cells. We showed that this activation was dose dependent on soluble factors in the conditioned media, as diluting the conditioned media treatment also resulted in a parallel reduction of the p-STAT3 (Y705) signal. Therefore, it is evident that p-STAT3-positive breast cancer epithelial cell lines and breast cancer associated fibroblasts secrete soluble factors, which can influence cells in a paracrine fashion.

The time frame for how this paracrine activation of STAT3 occurs was also interesting. We observed that increased STAT3 phosphorylation can occur in as little as 30 minutes, and this increased STAT3 phosphorylation can persist for at least 48 hours. After performing time point assays, we found that the phosphorylation level increased greatly at 30 minutes and reached a steady elevation at 6, 8, and 24 hours later but declined at 48 hours, although not to untreated levels. (Fig. 1D, Fig. 4D, and data not shown).

We next examined which factors were causing this increase in p-STAT3 activation. After performing ELISA analysis for various suspects, we found that an increase in p-STAT3 expression correlated with increased production of IL-6 in our breast cell lines. A growing body of evidence indicates a prominent and often contradictory role for IL-6 in breast cancer, and numerous reports have shown it to function in both a tumor-promoting and tumor-counteracting capacity [34]. These inconsistencies underscore IL-6's nature as a pleiotropic cytokine. IL-6 has been demonstrated as a potent activator of the JAK/STAT3 pathway [35], and has been shown to act in an autocrine manner to induce STAT3 to contribute to the pathogenesis of lung cancer [14]. In addition, mesenchymal stem cells, which are a major component of the fibroblast cell population in bone marrow, have recently been identified to secrete IL-6 in a paracrine fashion to enhance growth in ERα+ breast cancer cell lines [36]. Since bone is the primary site for metastasis in breast cancer patients, these results suggest IL-6 may be a major factor contributing to tumor growth and metastasis. The data presented in this manuscript demonstrate that IL-6 is a paracrine activator of STAT3 in ERα- breast cell lines as well. All of this data highlight IL-6 as a positive feed-back mechanism in STAT3 activation. STAT3 increases production of IL-6, which can further stimulate and prolong STAT3 activation in breast cancer epithelial cells in an autocrine as well as a paracrine fashion, as shown here in this manuscript. In support of IL-6 as one of the major paracrine factors leading to the activation of STAT3 in breast cells, we neutralized IL-6 from conditioned media or blocked a major component of its receptor (gp130). Blocking IL-6 or gp130 abolished the increased p-STAT3 seen in the MDA-MB-453 cell line treated with conditioned media. In a recent article by Berishaj, Gao, and Ahmed *et al.*, sequestration of IL-6 from MDA-MB-468 cells' conditioned media or blocking the gp130 receptor was sufficient to reduce conditioned media-induced STAT3 activation in MCF-10A cells [32]. Their findings and ours support each other, and further establish that IL-6 is a major contributing factor to the paracrine activation of STAT3 in breast cell lines.

However, the expected results were not observed when we treated MCF-10A cells with MDA-MB-231 cells' conditioned media. Neutralizing IL-6 in the conditioned media did not seem to significantly reduce p-STAT3. Nevertheless, blocking its major receptor, gp130, was sufficient to abolish this signal (data not shown). This could be due to one of several major possibilities: First, this could reflect that IL-6 is required at much lower concentrations to activate p-STAT3 in MCF-10A cells, and therefore, we were probably unable to completely block all of the IL-6 from the MDA-MB-231 cells' conditioned media using neutralizing IL-6 antibody. Second, IL-6 might not be the only soluble factor, which leads to the major activation of p-STAT3 in MCF-10A cells. It is also possible that another un-identified cytokine within the IL-6 family which signals through the gp130 receptor is also involved in paracrine activation of STAT3 in MCF-10A cells. We have yet to identify this other soluble factor that may be acting through the gp130 receptor. We have tried neutralizing LIF using LIF neutralizing antibody. However, neutralizing LIF in the conditioned media did not seem to reduce p-STAT3 (data not shown). The third possibility is that there is a combinatory effect between IL-6 and another factor, which leads to STAT3 activation. Further analysis should be made in this area to determine all the soluble factors, which should be targeted to sufficiently block paracrine activation of STAT3 in MCF-10A cells.

Next, it was important to determine if the cells acquired a transformed phenotype associated with this increase in p-STAT3 protein level. First, we examined the ability of soluble factors secreted by a p-STAT3-constitutively active cell line to upregulate cell proliferation of a non-cancerous cell line (MCF-10A). Soluble factors were able to cause an increase in cell proliferation rate in MCF-10A cells. We also saw increased levels of cyclin D_1 _which may explain the increase in cell proliferation. Since cyclin D_1 _is a downstream target of STAT3 it makes since that increased activity of STAT3 would lead to greater cell growth. This would play an important role in the transition of a normal cell into a cancer cell. There was also an increase in cell proliferation observed in MCF-10A cells and T-47D ERα+ breast cancer cells after treatment with BCF conditioned media (data not shown). When using MDA-MB-468 breast cancer cells as the source of conditioned media, we observed very limited enhanced growth as compared to the growth seen in MCF-10A cells treated with MDA-MB-231 conditioned media (data not shown). However, this correlates well with the greater level of IL-6 production from MDA-MB-231 breast cancer cells compared to MDA-MB-468 breast cancer cells. If IL-6 is the major factor contributing to this enhanced growth, then the much higher production of IL-6 by MDA-MB-231 breast cancer cells versus MDA-MB-468 breast cancer cells (Figure 2C) would account for the lack in enhanced cell proliferation effect in MDA-MB-468 breast cancer cells. When repeating this experiment with MDA-MB-453 treated with MDA-MB-231 cells' conditioned media, however, we were unable to observe an increase in growth phenotype. We attribute this to the fact that MDA-MB-453 is already a breast cancer cell line with increased growth rates compared to MCF-10A cells, and therefore did not acquire the phenotype of increased cell growth by MDA-MB-231 cells' conditioned media. However, since activated STAT3 may lead to several other carcinogenic mechanisms, such as resistance to apoptosis, angiogenesis, and tumor invasion [6], it is possible that paracrine activation of STAT3 in MDA-MB-453 cells may lead to those significant cancerous phenotypes. Therefore, it will be interesting to study additional phenotypic effects in the future so that we can fully understand how paracrine activation of STAT3 affects breast cancer.

Another useful phenotype which may correlate with STAT3 activation is chemoresistance. As with many human diseases, breast cancer patients may acquire chemoresistance to their drug therapies. Taxanes, including docetaxel, are often used for the treatment of breast cancer. However, chemoresistance or an incomplete response against this class of drugs is often acquired in patients [37]. Understanding which cellular mechanisms allow cancer tissues to acquire chemoresistance will help us to overcome this challenge.

In 2002, Real *et al. *described resistance to chemotherapy via overexpression of the anti-apoptotic protein Bcl-2 in metastatic breast cancer cells, which correlated with the activation of the STAT3 pathway [38]. As anti-apoptotic proteins have been found to be downstream targets of STAT3 activation in breast cancer [39], and chemoresistance in breast cancer cells has been linked to inhibition of apoptosis [40], it is likely that STAT3 activation may play an important role in chemoresistance in breast cancer. In fact, overexpression of activated STAT3 is found in most paclitaxel-resistant ovarian cancer cells, suggesting that this overexpression may lead to the resulting chemoresistant phenotype [41]. Increased levels of IL-6 have been shown to increase breast cancer cells' resistance to doxorubicin [42]. Our future experiments will look at a role of paracrine activated STAT3 as a source of chemoresistance in MDA-MB-453, MCF-10A, as well as other breast cell lines.

Our results further demonstrated that MCF-10A cells treated with conditioned media from MDA-MB-231 breast cancer cells can allow MCF-10A cells to produce and secrete their own IL-6. IL-6 can enhance motility of breast cancer cells [43] and autocrine production of IL-6 causes multidrug resistance in breast cancer cells [44]. Further, IL-6 is a potent growth factor for ER-α positive human breast cancer [36]. Our lab also found that after treatment of MCF-10A cells with MDA-MB-231 conditioned media, MCF-10A elevated their production of the Interleukin-8 (IL-8) cytokine by 5-fold (supplemental data not shown). Research has shown that IL-8 can modulate growth and invasiveness of breast cancer cells and the expression of IL-8 by human breast cancer cells correlates with bone metastasis *in vivo *[45,46]. While IL-8 is not a member of the IL-6 family, it is evident that paracrine signaling from breast cancer cell lines is important in transformation.

## Conclusion

We have shown that STAT3 can be activated through paracrine signaling in breast epithelial cells and this activation occurs through the JAK/STAT3 signaling pathway. IL-6 production is correlated with this increase in p-STAT3 and IL-6 appears to be one of the major factors contributing to paracrine activation. This paracrine activated STAT3 is important as it leads to transformed phenotypes, such as increased cell proliferation and an increase in production of IL-6. This suggests that IL-6 paracrine activation of STAT3 contributes to the process of breast tumorigenesis. Understanding which soluble factors are involved in this paracrine signaling and how these factors lead to STAT3 activation via the tumor microenvironment will provide new therapeutic targets for breast carcinomas and other cancers with elevated p-STAT3 levels.

## Abbreviations

STAT3: Signal Transducers and Activators of Transcription 3; VEGF: vascular endothelial growth factor; IL-6: Interleukin-6; BCF: breast cancer associated fibroblasts; p-STAT3: phosphorylated STAT3.

## Competing interests

The authors declare that they have no competing interests.

## Authors' contributions

JCL, SB, and BH participated in experiment designs, conducted the experiments, contributed to the analysis and interpretation of data, and drafted the manuscript. KS and BMH contributed to the interpretation and discussion of data. H-JL and TH isolated, characterized, and provided breast cancer associated fibroblasts as collaboration. JL contributed to overall experiment designs, research ideas, and preparation of the manuscript. All authors read and approved the final manuscript.

## Pre-publication history

The pre-publication history for this paper can be accessed here:


